# Learning to change a way of being: An interpretative phenomenological perspective on cognitive therapy for social phobia

**DOI:** 10.1016/j.janxdis.2010.03.018

**Published:** 2010-08

**Authors:** Freda McManus, Dawn Peerbhoy, Michael Larkin, David M. Clark

**Affiliations:** a University of Oxford Department of Psychiatry, & Oxford Cognitive Therapy Centre, Warneford Hospital, Oxford OX3 7JX, UK; b Department of Clinical Neuropsychology, Oxford Centre for Enablement, Windmill Road Headington, Oxford OX3 7LD, UK; c School of Psychology, University of Birmingham, B15 2TT, UK; d NIHR Biomedical Research Centre for Mental Health, South London and Maudsley NHS Foundation Trust and Kings College London, London SE5 8AF, UK

**Keywords:** Cognitive behavior therapy, Cognitive therapy, Social phobia, Patients’ experiences, Qualitative, Interpretive phenomenological analysis

## Abstract

Social phobia (SP) is a common and disabling condition for which cognitive-behavioral treatments (CBT) have demonstrated efficacy. However, there remains room for improvement. Hence, further exploration of the means by which CBT helps patients with SP is warranted. Studies examining patients’ perspectives on which aspects of treatment were most or least helpful may augment other established methodologies for identifying the more or less effective components and thus help to increase the efficacy and cost-effectiveness of CBT for SP. The current study used interpretive phenomenological analysis to analyze the transcripts of interviews with eight patients who had completed cognitive therapy (CT) for SP. Four related themes were identified: (i) *social phobia as a way of being*; (ii) *learning to challenge social phobia as a way of being: transformative mechanisms of therapy*; (iii) *challenges faced in the pursuit of change*; (iv) *a whole new world: new ways of being*. This analysis of patients’ experiences of CT for SP confirmed that the factors hypothesized to be important in maintaining SP in cognitive-behavioral models of the disorder are evident in patients’ descriptions of the processes of change in CT for SP (e.g., reducing internal focus of attention and reducing safety behaviors and avoidance). Helpful components of CT for SP were identified as areas where the protocol could be enhanced. Recommendations for the way in which CT for SP is implemented are made.

Social phobia (SP) is a common and disabling condition which, in the absence of treatment, typically follows a chronic course (Bruce et al., 2005; Kessler, Berglund, et al., 2005; Kessler, Chiu, Demler, & Walters, 2005) and is associated with marked impairment in social and occupational functioning (Erwin, Heimberg, Juster, & Mindlin, 2002; Stein & Kean, 2000). Considerable progress has been made in developing effective treatments for SP. Within psychological approaches, the best-validated treatments are behavioral and cognitive-behavioral. Several meta-analytic reviews have summarized studies comparing behavioral and cognitive-behavioral treatments with various control treatments and concluded that both are effective treatments for SP (Butler, Chapman, Forman, & Beck, 2006; Chambless & Hope, 1996; Fedoroff & Taylor, 2001; Feske & Chambless, 1995; Gould, Buckminster, Pollack, Otto, & Yapp; Taylor, 1996). Furthermore, individual studies report excellent maintenance of gains after completion of psychological treatment (e.g., Heimberg, Salzman, Holt, & Blendell, 1993; Liebowitz et al., 1999).

Although cognitive-behavioral therapies (CBT) have demonstrated efficacy in the treatment of SP, all trials of CBT have found that a significant proportion of patients remain symptomatic at the end of treatment and fail to reach an optimal level of functioning. Furthermore, these treatments consist of a complex set of procedures and while most would agree that both exposure and cognitive components contribute to the good outcome, there has been little investigation of the relative impact of different treatment components. Hence, further exploration of the means by which CBT helps patients with SP is warranted. Studies examining patients’ views on which aspects of treatment were perceived to be most or least helpful may complement other established methodologies (e.g., mediation analyses and component evaluation trials) for identifying the more or less effective components. This may help to increase efficacy and cost-effectiveness of CBT for SP by eliminating any unnecessary procedures or increasing the contribution of more effective procedures.

Patients’ perspectives have in general received limited attention in psychotherapy process or outcome research, and this shortfall seems particularly striking in CBT. However, involvement of patients in evaluating treatment is congruent with both the collaborative nature of CBT and with current mental health policy which emphasizes both the perspective of the patient/service-user and their involvement in research (Department of Health, 1999, 2006, 2007).

Although there have been no previous studies of patients’ experiences of CBT for SP it may be useful to consider what has been learnt from studies of patients’ experiences of CBT for disorders other than SP. Differing methodologies used and range of disorders studied present challenges in summarizing the existing literature (Hodgetts & Wright, 2007), however, at a basic level it is clear that patients report both positive and negative reactions to CBT interventions (e.g., Hodgetts & Wright, 2007; Laberg, Tornkvist, & Andersson, 2001; Messari & Hallam, 2003; Pain, C., Chadwick, P., & Abba, N., 2008; Pain, C.M., Chadwick, P., & Abba, M., 2008). Arguably the most consistent finding is that patients report both specific aspects of CBT interventions and non-specific factors (such as the therapeutic alliance) to be important contributors to the outcome. Other aspects frequently reported as helpful include the written formulation (Clark, Rees, & Hardy, 2004; Pain, C., Chadwick, P., & Abba, N., 2008; Pain, C.M., Chadwick, P., & Abba, M., 2008), increased self-awareness (Berg, Raminani, Greer, Harwood, & Safren, 2008; Clark et al., 2004) and testing things out/confronting fears (Clark et al., 2004; Nilsson, Svensson, Sandell, & Clinton, 2007). Some studies have also noted the influence of factors outside of the therapy on outcome such as continued employment and social support (Berg et al., 2008; Laberg et al., 2001). Regarding less helpful aspects or limitations of CBT interventions, several studies have reported patients’ preferences for a longer duration of treatment (Laberg et al., 2001; Clark et al., 2004; Berg et al., 2008), while Newton, Larkin, Melhuish, and Wykes (2007) note that the relational and social context in which patients make sense of their difficulties can conflict with CBT models. Some studies also highlight the importance of addressing patients’ initial attitude towards therapy which can be characterized by skepticism (Clark et al., 2004) pessimism (Pain, C., Chadwick, P., & Abba, N., 2008; Pain, C.M., Chadwick, P., & Abba, M., 2008), reservations (Berg et al., 2008) or unrealistic expectations (Mason & Hargreaves, 2001). Findings from these studies of patients’ experiences of CBT have been used to make recommendations about how CBT protocols could be further refined to enhance efficacy.

Alongside increasing recognition of the role of patients’ views in research and service planning (Hodgetts & Wright, 2007; Macran, Ross, Hardy, & Shapiro, 1999) there has also been increasing recognition of the utility of qualitative methods for understanding the experiences of service-users, and for capitalizing upon their insights, within a systematic and epistemologically coherent framework (Elliott, 2008; Hodgetts & Wright, 2007). For the most part, this work has developed within the experiential strand of qualitative psychology, because these approaches share a central concern with understanding the participant's point of view. Interpretative phenomenological analysis (IPA—see Smith, Flowers, & Larkin, 2009) is one such experiential approach, which has become well-established in applied psychology (Brocki & Wearden, 2006; Reid, Flowers, & Larkin, 2005). IPA draws upon key concepts from phenomenology, hermeneutics, symbolic interactionism and idiography, to focus upon the meanings which participants ascribe to events. It is particularly appropriate to the aims of this study because of its core epistemological commitment to insider accounts of “the human predicament,” (Smith et al., 2009, p. 5), and its integrative “capacity for making links between the understandings of research participants and the theoretical frameworks of mainstream psychology,” (Smith et al., 2009, p. 186).

To summarize, the current study uses qualitative methodology (IPA) to attempt to understand patients’ experiences during one of the established cognitive-behavioral treatments for SP, with the aim of understanding how different aspects of the treatment impact on the patients. It is hoped that this information may contribute to the further refinement of CBT protocols for treating SP. The particular treatment that is being studied is the cognitive therapy (CT) program developed by Clark, Wells and colleagues. This treatment has been show to be effective in four randomized controlled trials (Clark et al., 2003, 2006; Stangier, Heidenreich, Peitz, Lauterbach, & Clark, 2003; Mortberg, Clark, Sundin, & Aberg Wistedt, 2007) and compares favorably with treatment with SSRIs (Clark et al., 2003: Mortberg et al., 2007), exposure therapy (Clark et al., 2006), and group CT (Stangier et al., 2003; Mortberg et al., 2007).

## 1 Method

### 1.1 Ethical review

Ethical approval for the study was gained from the local NHS Research Ethics Committee.

### 1.2 Context of the study

The research took place in the broader context of the clinical work of the Centre for Anxiety Disorders and Trauma at the Maudsley Hospital in London. The Centre is an NHS service that offers evidence-based treatments (largely CT) to patients with anxiety disorders. The research arose out of a shared desire to better understand patients’ experiences of CT for SP so as to maximize the therapeutic impact of the treatment.

### 1.3 Participants

Service-users who had completed a standardized ‘CT for social phobia’ treatment protocol during the previous 2 years were identified by clinicians at the Center for Anxiety Disorders and Trauma. Exclusion criteria were (i) psychotic disorder, (ii) active suicidality, and (iii) lack of fluency in spoken English. Twenty-five people met these criteria and were invited to take part in the research by letter. Eight participants accepted the invitation.

The CT treatment protocol is described more fully elsewhere (Clark et al., 2003, 2006) and consisted of the following components: socialization to the model and reviewing a recent incident to draw out an idiosyncratic formulation according to Clark and Wells’ (1995) model; behavioural experiment(s) to evaluate the role of safety behaviours and self-focused attention in maintaining social anxiety; behavioural experiments involving video and other person feedback to correct distorted self-impressions (see McManus et al., 2009 for a more detailed description of these procedures); training in externally focused attention; integrated cognitive restructuring and behavioural experiments to re-evaluate and test fearful predictions; re-scripting of early, socially traumatic memories linked to negative self-imagery; Wild, Hackmann, and Clark (2008) for a fuller description; addressing anticipatory and post event processing; cognitive work on residual negative self appraisals; and relapse prevention planning.

All therapists had experience of delivering CT for SP in NHS settings and had regular supervision (group and individual) to ensure high fidelity to the treatment protocol.

### 1.4 Measures

In order to situate the sample in terms of their levels of psychological distress, and their comparability to other groups of service-users, participants completed the following measures: the Beck depression inventory (BDI: Beck, Ward, Mendelson, Mock, & Erbaugh, 1961), Beck anxiety inventory (BAI: Beck, Epstein, Brown, & Steer, 1988) and the social phobia anxiety inventory (SPAI) (Turner, Beidal, Dancu, & Stanley, 1989). Socio-demographic and clinical information about participants is provided in Table 1.

As can be seen from Table 1, participants received between 10 and 18 90-min CT sessions which included both weekly sessions and monthly follow-up appointments. Participants had completed treatment a mean of 11.3 months (SD = 2.8) prior to the interview. Prior to beginning treatment, all participants met criteria for a diagnosis of SP as assessed by the anxiety disorders interview schedule (ADIS-IV, DiNardo, Brown, & Barlow, 1995) but after treatment four of the eight participants no longer met diagnostic criteria. At 1 year follow-up six of the eight participants no longer met diagnostic criteria for SP. Participants’ scores on the SPAI reduced from a mean of 129.95 (23.17) prior to treatment to 68.68 (43.90) following treatment and 70.27 (33.88) at 1 year follow-up. Beidel, Turner, Stanley, and Dancu (1989) suggest a clinical cut-off score of 80 on the SPAI. From Table 1 it can be seen that participants’ mean SPAI score was above this cut-off prior to treatment but not after treatment, and only Sarah and Peter's scores remained above the cut-off at 1 year follow-up. Similarly, participants’ scores on the BDI and BAI were in the non-clinical range post-treatment (means of 3.87 (4.56) and 7.13 (6.24), respectively) and at 1 year follow-up (means of 3.00 (3.89) and 6.00 (6.36), respectively).

### 1.5 Data collection

A semi-structured interview schedule was prepared in accordance with the recommendations of Smith and Osborn (2003). Questions were open-ended, and designed to invite participants to narrate, and then reflect upon, their experiences of CT for SP. They were asked how they came to have CT for SP, what they recalled about the experience, which if any aspects of treatment they found helpful or unhelpful, what if any impact CT had on their SP, and about any other influences that impacted on their SP during this time. Interviews were carried out by DP, at the NHS service where participants had received their treatment. The schedule was used as a flexible guide only, as appropriate (Smith et al., 2009). Interviews lasted between 45 and 60 min. They were audio recorded, and all semantic content was transcribed in full and anonymised at point of transcription.

### 1.6 Data analysis

Standard analytic procedures for IPA were followed, which have been reported in detail elsewhere (e.g., see Smith & Osborn, 2003; Smith et al., 2009).

The second author [DP] took the lead in developing a close, line-by-line analysis of the experiential claims, concerns, and understandings of each participant, and in the subsequent identification of the emergent patterns within this experiential material across the data set. At each stage these were discussed and developed through detailed collaboration with the third author [ML]. The final thematic structure for these patterns was developed through discussion between DP, ML and FM.

#### 1.6.1 Credibility

Several steps were taken to enhance methodological rigor and the credibility and trustworthiness of the analysis (Elliott, Fischer, & Rennie, 1999; Smith & Osborn, 2003). In addition to the various collaborative processes described above, further supervision for the analysis was provided by a peer supervision group, which enabled the accounts to be subjected to a broader perspective and facilitated credibility checking across themes.

## 2 Results

### 2.1 Qualitative analysis

Four super-ordinate themes were identified, each with its own sub-structure of further sub-ordinate themes (see Table 2).

Each of the super-ordinate themes was supported by evidence from all transcripts. For purposes of brevity, we have included a limited selection of the many available exemplar quotes.

#### 2.1.1 Social phobia as a way of being

Although the first theme does not directly relate to participants’ experiences of CT for SP, it is briefly discussed to clarify and contextualize participants’ experiences of CT, by illustrating their shared understanding of SP, which provides a context for their perceptions of receiving CT for SP.

Participants described both stressful life events, and their character (being a shy and introspective person more generally) as having a role in the onset of their SP. For all participants there was a sense that SP had permeated every aspect of their lives, and become a way of being. The key characteristics of this way of being included a sense of detachment – all participants spoke of being introspective and absorbed in their own world, with difficulty looking outside of themselves – that impacted on their relationships in all areas of life. They described the quality of this ‘internally focused’ self-absorption as overwhelmingly negative. For example, Tom described how he changed from being a confident individual to someone that he perceived to be “stupid, thick, weak.” All had a sense that something was wrong, but (with only one exception) they were uncertain about the cause of the problem, and afraid of the consequences of finding out more. These difficult emotions were compounded by the powerful embodied experience of the anxiety.

A recurring theme was the strong sense of shame and embarrassment felt at experiencing the intense, uncontrollable anxiety. Alongside the sense of shame were experiences of being watched and judged by the outside world (e.g., “I thought people would criticize me or judge me or push me away” Gina), and an attempt to manage these difficulties by keeping them secret, and where possible, avoiding the social world altogether (e.g., “I suppose I started avoiding a lot of things, asking people to do things for me” Susie). Collectively then, participants shared an understanding of SP as powerfully unpleasant experience, which isolated them from other people, encroached upon all aspects of their being, and had a negative impact upon both their mood and their perceptions of self and others.

#### 2.1.2 Learning to challenge social phobia as a way of being: transformative mechanisms of therapy

This theme arose from participants’ reflections on the personal impact of CT and focuses on participants’ experiences of those mechanisms of therapy which they felt to be transformative for their difficulties. All participants described how important the *relationship with the therapist* was in enabling them to be open about their difficulties. Participants felt ashamed of their SP, so the perception of the therapist's openness *and* expertise appeared to increase their trust and put them at their ease:

“I felt like she wasn’t looking down on me in any way, which was quite important I think because I guess it's obvious really but you know, you feel sort of, felt embarrassed you’ve got it” (Tom).

“He was very gentle and it was nice to finally speak to somebody who specializes in social phobia, I’ve seen other people who have got no idea really, I had to explain to people, me telling them how I feel. I felt comfortable saying to someone who knows stuff, so yeah I found it quite helpful” (Susie).

As these extracts demonstrate, trust and belief in the therapist, and in the therapists’ skills, were central to many of the positive experiences which participants reported. The importance of liking the therapist as a person resonated throughout the transcripts; understanding, validation and support were perceived to be paramount.

Seven participants also expressed relief that there was a ‘label’ for their difficulties. They spoke of the therapeutic *value of having a diagnosis and formulation*, and described how this enabled them to better understand their difficulties, and ultimately, to view themselves more positively:

“I think it's really like opened my mind and it was really, really useful because first of all I realized that I wasn’t the only one … I didn’t feel like I was not normal, this can happen to anyone. So from there on I felt I was a bit more confident … and positive” (Gina).

As this extract illustrates, the confusion which some participants expressed about the source of their difficulties was ameliorated by the information provided by the therapist. Similarly normalizing effects were described in relation to participants’ feelings of embarrassment and shame about their difficulties. Participants valued the formulation as a non-blaming explanation of how their difficulties had developed, and were maintained. They felt that the formulation helped them to understand the predisposing factors and causes of their SP, its triggers and its maintaining cycle. In particular, they became aware of how the different components of the problem interacted with one another, in a cyclical process that maintained the anxiety. They also valued having this information presented to them visually in the form of a diagram:

“We discussed where it might have come from and my kind of low self-esteem when I was an adolescent, and things like that … and we jotted down a kind of flow diagram, almost of how it reinforces itself and how the physical interacts with the mental and how it's a big kind of feedback cycle … I suppose yeah it was useful to look at this in a more objective way … yeah so that was useful” (Sarah).

While seven of the eight participants spoke of the value of the formulation, one expressed reservations. His experience of drawing out the formulation had not felt like a collaborative venture: “She just got a diagram out and said this is a model of what's going on” (Tom) and he struggled to understand or remember it, but was too embarrassed to say so at the time. He felt that his anxiety may have inhibited his ability to focus on it and would have preferred to have a simpler model or have the components added in stages.

Finally, all participants described how they had *learned to interpret their own experiences differently, through the experiential work practiced in therapy*. They described how this experiential work involved an alternative cycle: predicting and testing possibilities; learning to shift their focus away from their internal state, and towards external events; learning that how one appeared to others might not be a direct reflection of how one felt—and learning to identify their avoidance and safety behaviors. Instead, they described how engaging in anxiety-provoking social situations had enabled them to specify and test predictions about what might happen. They commented on the value of first, articulating specific predictions about what might happen in the situation, and second, testing out those predictions:

“Writing down what you are thinking when you are thinking those negative thoughts, and trying to look at it objectively and assess, objectively if it's a valid thing to think and most of the time you are going to think that you are just crazy, but actually it's not that bad” (Sarah).

“I think it was quite useful to be able to write down your situation and you predict that situation and how anxious you would be while you are in the situation - you realize that you are not so anxious as you thought you were going to be. So that's like opened your mind so you can see it's not what you thought, it's not so scary maybe as I thought. So being able to write it down, you know, helps” (Gina).

As in Sarah's account, above, participants described how the hypothesis-testing approach allowed them to see that their predictions about what might happen in social situations were exaggerated. Such predictions were tested via behavioral experiments which involved viewing themselves on video, or getting feedback from observers. Consequently, participants described reconsidering their beliefs about how anxious they would appear or feel in such situations, as illustrated in Gina's account, above. Thus, one aspect of such learning was the revelation that how one *appeared* might *not* reflect how one *felt*:

“While you can feel very nervous on the inside you don’t generally look as nervous on the outside. And also that it's not really that important, you know, whether, if you are a bit nervous, that's not a crime or anything bad, it's just that you’re a bit nervous and you know, that's nothing to really hide, yeah” (Lucy).

A corollary of this distinction between external appearance and felt experience was evident in further experiential work which was perceived to help participants to shift their focus of attention from the internal to the external. This skill was practiced in therapy sessions and in social situations as homework:

“My therapist just got me to listen to the sounds and stuff and to focus on those during the session, so I was closing my eyes and listening to the sounds and then just looking for some colors as well. That's what we did during the session and I did the same sort of things myself outside the sessions” (Peter).

These lessons appeared to be transferred from therapy to other realms. Participants described social situations both in and outside of sessions, and reflected that through ‘facing the fear and doing it anyway,’ they had learned that their beliefs about what they thought would happen were always much worse than what actually occurred:

“I think yeah the most helpful thing for me was having to do the things that I’m scared of and just realizing that I’m not going to die” (Susie).

Observations of others provided further learning experiences. For example, five participants described how they had learned that others were somewhat oblivious to their anxiety and were not as judgmental as they feared:

“She started to shake as well while having soup. There was this person in front of her, and I was looking at both of them and she told me afterwards, she asked me ‘Was this person talking to me and interested in what I was saying or was she looking at my hands shaking?’ ‘She wasn’t looking at her hands shaking she was looking at you,’ so I realized that maybe if you shake, it doesn’t matter to people, people are not going to make much out of it” (Gina).

As in Gina's account above, many participants commented on the value of soliciting feedback from others on how they came across in social situations. However, five participants questioned the validity of the feedback they received on their apparent anxiety, in group-work and on video. They questioned the independence of the raters or whether the quality of the video was good enough for them to draw definitive conclusions. They therefore doubted some of the positive feedback on their performance:

“They’d still give a slightly positive spin on anything they’d say to make you feel better about yourself” (Peter).

Seven of the eight participants also described having learnt that their old avoidance and safety behaviors actually did not help them to keep feel better, but instead exacerbated and maintained their anxiety (“Avoiding situations you don’t like only made it worse,” Tom). They emphasized the value of practicing and repeating the strategies that they had acquired in therapy, instead (“We kept on this theme every week and eventually you think, ‘Ok yeah that sinks in,”’ Tom). Participants described how they continued to use their new skills to specify and test out negative predictions after therapy ended, and felt that it continued to be useful (“I still use it when I have moments” Gina).

To summarize, participants described the process of learning to change their way of being in the world, via a series of social scenario experiences which were designed, and given particular meanings, in the context of the work undertaken with their therapists. This process although clearly challenging, was also experienced as positive, liberating and transformative.

#### 2.1.3 Challenges faced in the pursuit of change

This theme explores some of the challenges presented by CT, and the process described above, in more detail. All participants commented on the ‘*emotional roller-coaster*’ that characterized their experience of the therapy process. Participants reported feeling hopeful about change (Sarah, Susie, Jack), but also felt hopeless or a sense of failure when setbacks were experienced (Ruth, Sarah, Gina, Susie). Some commented on experiences of strong anxiety (Sarah, Tom, Susie), and relief, when a goal had been achieved:

“I felt very good … because I had done something that I was scared of and it wasn’t as bad as I thought and it felt like, after the session, I felt like yes I can do things, I can actually conquer it and learn to live with it” (Susie).

The tropes of mastery (‘conquer it’) and self-improvement (‘learn to live with it’) were common, but participants also described some ambiguity and ambivalence, which challenged this narrative of triumphant personal development. Tom, for example, reflected that although he felt positive after sessions, he also felt confused and bewildered at times:

“Yeah, I felt good normally if I’d done good stuff and sometimes I felt confused if I didn’t entirely understand what had happened, for instance we might have gone through that model thing or something. I might have come out feeling a bit bewildered and confused but generally I felt pretty good, yeah” (Tom).

Three participants also described the *difficulty of changing long-established habits in a short space of time*, even with insight and motivation. Susie, for example, found it difficult to give up her safety behaviors, and Gina struggled to use external focusing strategies:

“The most difficult was those where I had to concentrate on what was going on around me, because I haven’t done that all my life, because I’ve been very inwards thinking about my feelings and my emotions, so it was very difficult to break that pattern and to try and concentrate on others … but it kind of helps to remind myself to use these strategies which didn’t come naturally to me because being so many years behaving in a certain way, you have to make an effort to change, to change your behavior” (Gina).

The underlying understanding that engagement in therapy is a form of moral and motivated self-improvement (‘you have to make an effort to change’) is striking here in Gina's account. All participants drew upon an understanding of therapy as a source of *tools* (for learning and change), and of patients as the engine for that change, all in the context of collaborative *work* with the therapist. This is a fairly common conceptualization of therapy, and is not unique to CT. It does have some potential implications for patients who do not progress as quickly as they might hope, however. It is important to note that six participants (Sarah, Tom, Jack, Ruth, Peter and Lucy) described feeling they needed further sessions to consolidate their gains. From Table 1 it can be seen that on average participants maintained the gains they had made in therapy when assessed at follow-up, a finding that is also observed in all four RCTs of the treatment (Clark et al., 2003, 2006; Mortberg et al., 2007; Stangier et al., 2003). However, this average is made up of both individuals who show further improvement and individuals who show some deterioration. Consistent with this, Ruth and Tom commented that while they had made gains in treatment, they felt that they had lost some of those gains since treatment ended. Sarah reported that her social anxiety and sociability had continued to improve since finishing CT—but she attributed this to medication, rather than CT:

“I didn’t think it [CT] went far enough, you know, it kind of almost scratched the surface and I was starting to do things that I found really problematic but then it stopped” (Sarah).

Three participants spoke of the challenges involved in *transferring theory to practice*, and in generalizing to the ‘real world’ outside of the sessions. Sarah, for example, commented that she found it difficult to specify predictions to test outside of sessions, as many of the things she wanted to test felt intangible; Tom explained that he found it difficult to break down and analyze his thoughts; Susie felt that her level of anxiety in the therapy scenarios was not as high as her level of anxiety in real life situations, which made it difficult to employ the same strategies outside of sessions:

“Sometimes I felt that the situations that I was nervous about and were created in the session, I was less nervous in the same situation, than I was in that situation outside of the sessions. Outside of the sessions I’d be 10 times as bad and nervous … it was harder on my own, so sometimes I can’t do it” (Susie).

Tom and Sarah both reflected they had difficulty learning and consolidating skills during treatment. Tom, for example, commented that he felt overloaded with information, and Sarah said that at times her anxiety was too overwhelming during treatment and that she only was able to use the strategies after finishing treatment, once her anxiety was treated with medication. Similarly, two participants felt that the blueprint completed at the end of therapy was a useful tool and three described how it had acted as a refresher, and a mood enhancer, when times were difficult. But five participants reported having largely forgotten about the blueprint and thus not finding it a particularly helpful tool.

Here we have seen that there is a counter-point to the positive experiences and changes reported in Section 2.1.2. For seven participants the process was emotional and challenging, and at times uncertain. Sarah, who showed only modest clinical improvement (see Table 1), also expressed some dissatisfaction with CT at a conceptual level, feeling it was somewhat unrealistic or simplistic in its aims:

“I think I had a bit of a problem with CT in that the way I understood it, it's kind of saying, you know, none of that matters, it's fine you know, ‘Just do your experiments and you will realize that.’ Although I thought it was a good way to think, it felt there was an element of CT that I couldn’t believe in, because it didn’t seem realistic” (Sarah).

#### 2.1.4 A whole new world; new ways of being

All participants described some *relief from, and re-appraisal of, anxiety*. All spoke of a reduction in the overwhelming physical symptoms of anxiety, and in preoccupation with those symptoms, since having CT. While participants still experienced some anxiety in certain social situations, they felt this was now more manageable and no longer all consuming. They perceived their anxiety to be within the normal range and not to have the life-limiting effects that it had prior to therapy. For example:

“I still get nervous, I would still call myself shy, but it's not as bad as it was before, now I can now go into situations whereas before I would just avoid it, and now I know I can get through them” (Susie).

Participants reflected that they had learnt to accept their anxiety and had an increased sense of control over their anxiety. They also spoke of greater acceptance of their anxiety as a consequence of feeling less shame about it (“Everyone has you know, certain fears,” Jack).

Resonating throughout all narratives was the experience of the impact of CT in prompting greater *acceptance of themselves and others*, following treatment. Participants described how their self-image had become more positive. Five participants spoke of a newfound compassion for themselves, and of wanting to be kinder to themselves:

“But, also not to punish yourself if you don’t feel like doing something. Take one thing at a time, small steps, you can’t do everything. If you need time then you need to respect yourself a bit more” (Ruth).

As a result of learning that others were not judging them, four participants reflected that they felt more accepted in society, and had learnt that they could enjoy life, even if they showed visible anxiety.

“It's just a different feeling, a different wellbeing really that you know that life's about, that people aren’t always judging you … life's there to be enjoyed, if you put a foot wrong it's not the end of the world. You know, you just take the pressure off yourself just knowing those things” (Jack).

This greater acceptance of themselves and others as less judgmental may have contributed to participants unanimously reporting *re-engaging with the world*, having become more socially active since having CT. Seven of the eight were working or studying at the time of the interview and some had changed jobs since having CT. All spoke of reduced avoidance of social situations, and initiating new social ventures. Lucy described taking more risks and trying new ventures, and thus felt that she was consolidating gains and continuously learning:

“Yeah I think lots of things changed … I try to take more risks … just trying new things that you don’t try before, like, mostly be yourself. I just am doing more things like, going for walks with people that I didn’t know was really good as well, going for a class that I like, try to find new ways to improve all the things that I was learning, put everything together” (Lucy).

Participants also described another form of reduced avoidance and renewed engagement with the world—taking the risk of revealing their true selves, including their SP, to others:

“Whereas in the past I would have felt like um there's something to discover about me which is you know undesirable sort of thing, um, that isn’t there anymore, so even if I am self conscious, It's just like I’m a normal bloke so it's not a big deal really” (Jack).

## 3 Discussion

### 3.1 Summary of themes

The analysis identified four related themes. This first theme, ‘*social phobia as a way of being*’ provided a background to the changes that occurred during therapy by describing the participants’ experiences of living with SP prior to seeking treatment. The second theme, ‘*learning to challenge social phobia as a way of being: transformative mechanisms of therapy*’ exemplified participants’ experiences of learning to challenge their SP and their impressions of the transformative mechanisms of CT for SP. The third theme, ‘*challenges faced in the pursuit of change*’, shows some of the ways in which participants struggled to make the changes in CT for SP. The final theme ‘*a whole new world: new ways of being*’ focuses on the on-going changes that participants experienced as a result of the changes made during therapy.

### 3.2 Social phobia as a way of being

The narrative here largely describes the participants’ sense that their SP was a debilitating problem that affected every aspect of their lives and often formed part of their self-concept. This view of the disorder as being part of their self-concept may help to explain the strong sense of shame and embarrassment felt by patients with SP and their reluctance to seek treatment for the disorder (Kessler, Olfson, & Berglund, 2003). Participants’ sense of the pervasiveness and persistence of the disorder is confirmed by research showing that SP is a debilitating disorder that, in the absence of treatment, tends to run a chronic course (Kessler, Berglund, et al., 2005; Kessler, Chiu, et al., 2005; Stein & Kean, 2000).

### 3.3 Learning to challenge social phobia as a way of being: transformative mechanisms of therapy

As in other qualitative studies of patients’ experiences of CT the value of the therapeutic relationship in enabling change is highlighted. Participants particularly valued feeling accepted by the therapist, and the normalizing effect of the therapist's experience of SP. A sound therapeutic relationship may be especially important in psychotherapy for SP to enable patients to be open with their therapists about their difficulties. This may confirm Beck, Rush, Shaw, and Emery's (1979) suggestion that the therapeutic relationship is “necessary but not sufficient” to produce change in CBT (p. 45).

Seven of the eight participants reported the process of collaboratively drawing out the formulation to be a positive experience, in that it helped them to make sense of how they had developed the problem and to view their SP as a problem that was affecting them, rather than as a personal characteristic or failing. Drawing out the formulation was experienced by most as a normalizing and de-stigmatizing experience. This is a more consistently positive report than in a previous qualitative study looking at the impact of formulation in CBT for psychosis (Pain, C., Chadwick, P., & Abba, N., 2008; Pain, C.M., Chadwick, P., & Abba, M., 2008). The more positive reactions to formulation in the current study warrant further investigation. It may be that formulation is a more consistently helpful exercise in CT for SP than it is in CBT for psychosis, possibly because in SP it serves a normalizing and de-stigmatizing function, whereas the diagnosis of a psychotic illness is associated with greater stigma (Mann & Himelein, 2004). Or, as highlighted by Tom's comments above, it is important for the formulation to be a collaborative venture and in psychosis there is more likely to be a disparity between the therapist and patient's views of the problem. It is also worth noting that in the current study the therapists were all highly trained and specialized in treating SP with CT so it may be that they had higher than average levels of skills in drawing out cognitive-behavioral formulations.

Experiential learning was a resounding theme in participants’ descriptions of the transformative mechanisms of CT for SP. They particularly emphasized the value of specifying and testing out predictions about how they may come across to others, of learning to shift their focus of attention in social situations, and of reducing avoidance and safety behaviors. These findings confirm the value of the experiential components of CT and effectiveness of behavioral experiments in treating SP (McManus et al., 2009). An important end product of this work seemed to be that participants learned that they did not come across as badly as they feared, and that the ‘cost’ of negative social outcomes was not as high as they had anticipated (i.e., even when it did go wrong, they coped). They described learning to shift the focus of their attention away from themselves and towards what was happening in the social situation. They were then able to identify and reduce safety behaviors and avoidance, and to put themselves in situations that would test their negative predictions.

Participants particularly valued the emphasis on conducting multiple behavioral experiments and other experiential exercises. This is consistent with findings from Mason and Hargreaves’ (2001) qualitative study of patients’ experiences of mindfulness-based cognitive therapy for depression, in which participants noted the value of consistently practicing techniques for bringing about therapeutic change. The patients’ valuing of repeated experiential work is important in considering recent suggestions that therapists may avoid, or prematurely abandon, carrying out therapy tasks that involve exposure or behavioral experiments (Becker, Zayfert, & Anderson, 2004; Schulte & Eifert, 2002; Waller, 2009).

One challenge for the participants as they tested out how they were perceived by others was their inclination to doubt the evidence generated by therapy. Understandably, some participants questioned validity of feedback from the therapist because it lacked neutrality—the therapist clearly wanted to make the patient feel better. A common technique to overcome this possible bias is for the therapist to video the patient in social interactions and let the patient judge for themselves how they came across. However, some participants reported not trusting the quality of the video image to provide disconfirmation of their fears. This suggests that therapists may need to spend more time setting up video equipment in order to ensure that patients are satisfied that it can accurately detect the behaviors that are the focus of their concerns. Further Socratic discussion of patients’ doubts may also be beneficial. The key end product of much of the experiential work seemed to be, by any combination of methods, the patient learning that their self-impression is overly negative (Rapee & Lim, 1992) and thus, that they do not come across as badly as they feel they do. An on-going challenge for CT therapists is to employ creativity in working collaboratively with the patient to design therapy tasks that generate evidence that is perceived by the patient as relevant to their concerns and valid and credible.

### 3.4 Challenges faced in the pursuit of change

Participants reported feeling that CT was, at times, an emotional roller-coaster with ups and downs. The positive changes were welcome and exciting but setbacks and perceived failures were equally emotional. Propensity of CT for SP to trigger negative feelings is likely to put patients at risk of drop-out. Therapists providing CT for SP must take care to adequately prepare patients for setbacks and to ensure that a sound therapeutic relationship, in which the patient can openly expresses doubts and give negative feedback, is in place before experiential work is undertaken. That said, the four clinical trials of CT for SP reported low drop-out rates (5% on average), at least when the treatment is delivered by experienced therapists.

The theme of repeated experiential work was reiterated as participants noted the difficulty of changing long-established habits in a short space of time and of generalizing from the therapy setting to the real world. Consistent with other studies of participants’ experiences, some participants would have preferred a longer duration of treatment (Berg et al., 2008; Clark et al., 2004; Laberg et al., 2001) to consolidate the changes made. Future research might usefully assess whether such individuals would show further clinical gains if therapy were extended. It is also worth noting that the ‘blueprint’, a component of CT explicitly intended to help patients use their CT skills after treatment has ended, did not seem to have been used by some participants even though it may have been needed. One question worth consideration for future research may be to identify how CT could have a greater impact on helping patients with SP to further generalize the skills they learnt in therapy. Clinical experience suggests that ensuring as much of the experiential work as possible takes place in situations that are similar to the patient's ‘out of therapy’ environment is helpful. That is, using therapy time for the therapist to accompany the patient into real world settings to carry out the experiential components of CT. For example, going into local shops, restaurants, etc., in order to carry out behavioral experiments or to practice focusing externally (Hirsch & McManus, 2007). Homework tasks represent another opportunity for therapists to bridge the gap between therapy sessions and the patient's real world (Kazantzis, Deane, Ronan, & L’Abate, 2005).

### 3.5 Whole new world: new ways of being

Participants described the impact of CT for SP as a welcome relief from the overwhelming physical symptoms of anxiety. A further major change seemed to be acceptance: participants described an acceptance and normalizing of their anxiety, with the consequent expectation that others would be more accepting of it too. So not only were they experiencing less anxiety but they were also less anxious about experiencing anxiety, and about others noticing their anxiety. There seemed to be a ‘virtuous circle’ of participants being more self-accepting of their social anxiety and of themselves as people, and also more accepting of others as less critical and judgmental. It seems likely that experiential work testing out others’ reactions to, for example, showing anxiety symptoms, may have contributed to this greater acceptance of self and expectation of acceptance by others.

In discussing the positive changes arising from CT for SP participants also cite reduced avoidance and re-engaging with the world. This was both in terms of overt situational avoidance but also in terms of taking more risks interpersonally.

## 4 Conclusions

Conclusions drawn from this study must be interpreted in the light of the limitations of the study—in particular, the small sample size and the high proportion of treatment responders, and the focus on only one version of CT for SP. The results confirm some of the helpful components of CT for SP, and identified areas where the protocol might be enhanced. The factors hypothesized to be important in maintaining SP in cognitive models of the disorder (e.g., Clark & Wells, 1995; Rapee & Heimberg, 1997) are evident in participants’ descriptions of the processes of change in CT for SP (e.g., reducing internal focus of attention and reducing safety behaviors and avoidance). There is also a sense of greater acceptance, not only of their anxiety but also of themselves and others. This theme of ‘acceptance’ is consistent with recent developments in the broader field of CBT (e.g., Hayes, Folette, & Linehan, 2004) and has been echoed in other studies of patients’ experiences (Allen, Bromley, Kuyken, & Sonnenberg, 2009) but as yet is not explicitly addressed in CT for SP. Results from this study confirm the value of the experiential ‘testing out’ component of CT in overcoming SP but also note that care must be taken that the data generated are believable and seen to generalize to the patient's world outside of therapy. Similarly, the value of drawing out a diagrammatic formulation is noted but again care must be taken to ensure that it is a collaborative process at the patient's pace.

## Figures and Tables

**Table 1:** Participants’ age, ethnicity, employment status, number of sessions, and scores on the social phobia anxiety inventory (SPAI) at pre-treatment, post-treatment and follow-up.

Participant	Age	Ethnicity	Employment status	No of sessions	SPAI score		
					Pre	Post	Follow-up
Gina	38	White European	Unemployed	8 + 3 follow-up	152.5^a^	13.0	21.5
Tom	30	White European	Student	7 + 3 follow-up	117.8^a^	22.3	65.5
Jack	31	White European	Employed	8 + 3 follow-up	127.0^a^	88.2^a^	78.4
Lucy	41	Other	Employed	15 + 3 follow-up	116.9^a^	20.3	41.3
Ruth	36	Other White	Student	14 + 3 follow-up	109.8^a^	76.0	63.2
Susie	23	White European	Employed	14 + 3 follow-up	106.1^a^	97.4^a^	62.1
Peter	25	White European	Employed	7 + 3 follow-up	173.7^a^	124.2^a^	132.3^a^
Sarah	26	White European	Employed	7 + 3 follow-up	135.8^a^	108.3^a^	98.0^a^

Mean (SD)	31.25 (6.54)	n/a	n/a	13.0 (3.63)	129.95^a^ (23.17)	68.68 (43.90)	70.27 (33.88)

For all patients at pre-treatment and for 7 patients at post-treatment/follow-up diagnosis is based on the ADIS. One patient (Ruth) was not interviewed at post-treatment/follow-up but fell below the clinical cut-off on the SPAI at both times.

a Met criteria for diagnosis of SP.

**Table 2:** Super- and sub-ordinate themes arising from analysis of participants’ transcripts of their experiences of CT for SP.

Super-ordinate themes, indicated by bold font in the text	Sub-ordinate themes, indicated by italic font in the text
2.1.1 Social phobia as a way of being	
2.1.2 Learning to challenge social phobia as a way of being: transformative mechanisms of therapy	Value of the therapeutic relationship. Value of diagnosis and formulation. Learning to interpret experiences differently through experiential learning practiced in therapy.
2.1.3 Challenges faced in the pursuit of change	Therapy being an emotional roller-coaster. Transferring theory and skills to practicing in the real world.
2.1.4 A whole new world: new ways of being	Relief from and reappraisal of anxiety. Enhanced acceptance of anxiety and of self and others. Re-engaging with the world.
